# Signs of Selection in Synonymous Sites of the Mitochondrial Cytochrome b Gene of Baikal Oilfish (Comephoridae) by mRNA Secondary Structure Alterations

**DOI:** 10.1155/2015/387913

**Published:** 2015-05-31

**Authors:** Veronika I. Teterina, Anatoliy M. Mamontov, Lyubov V. Sukhanova, Sergei V. Kirilchik

**Affiliations:** Limnological Institute, Siberian Branch, Russian Academy of Sciences, P.O. Box 278, Irkutsk 664033, Russia

## Abstract

Studies over the past decade have shown a significant role of synonymous mutations in posttranscriptional regulation of gene expression, which is particularly associated with messenger RNA (mRNA) secondary structure alterations. Most studies focused on prokaryote genomes and the nuclear genomes of eukaryotes while little is known about the regulation of mitochondrial DNA (mtDNA) gene expression. This paper reveals signs of selection in synonymous sites of the mitochondrial cytochrome b gene (Cytb) of Baikal oilfish or golomyankas (Comephoridae) directed towards altering the secondary structure of the mRNA and probably altering the character of mtDNA gene expression. Our findings are based on comparisons of intraspecific genetic variation patterns of small golomyanka (*Comephorus dybowski*) and two genetic groups of big golomyanka (*Comephorus dybowskii*). Two approaches were used: (i) analysis of the distribution of synonymous mutations between weak-AT (W) and strong-GC (S) nucleotides within species and groups in accordance with mutation directions from central to peripheral haplotypes and (ii) approaches based on the predicted mRNA secondary structure.

## 1. Introduction

Recent studies have shown a significant role of synonymous sites in the regulation of gene expression and fitness [1–5]. Synonymous mutations influence not only the rate of translation but also posttranslational modification of proteins [6]. One form of such regulation involves alterations of the secondary structure of messenger RNA (mRNA). The impact of synonymous codon sites on mRNA stability, folding, and, consequently, gene expression is greater than the impact of nonsynonymous codon sites [4, 7] and can therefore be exposed to various forms of natural selection more than previously believed. To date, most studies of the effects of synonymous mutations on gene expression have focused on prokaryote genomes and the nuclear genome of eukaryotes, while virtually little is known at the mitochondrial DNA (mtDNA) level. Meanwhile, the identification of signs of selection in synonymous sites of mitochondrial genes in natural objects can be an area of focus for understanding these processes. Baikal oilfish can be considered such an object.

Big golomyanka (BG) or big Baikal oilfish (*Comephorus baicalensis*) and small golomyanka (SG) or little Baikal oilfish (*Comephorus dybowskii*) are the only representatives of the endemic genus* Comephorus* of the Comephoridae family of Lake Baikal cottoid fish [8]. The habitats of these species largely overlap; representatives of BG and SG are distributed throughout Lake Baikal with the exception of the coastal zone and are found at all depths of the lake. Baikal oilfish are the most abundant fish in Lake Baikal. According to Starikov [9], the numbers of SG and BG were approximately 22.2–41.2 and 7.1–10.8 billion specimens, respectively, for the period of 1969 to 1972. These species are very adapted to the pelagic lifestyle of an open lake. This is reflected in the fact that golomyankas are extremely sensitive to changes in environmental conditions. The optimum water temperature for these species is ~3.6°C. During experiments when the temperature rose above 8.0–8.5°C, BG instantly fell into a stupor and began to die [10]. Both species are viviparous. BG breeds throughout the year, with two peaks of spawning in August, September, and December [11–13].

**Figure 1:**
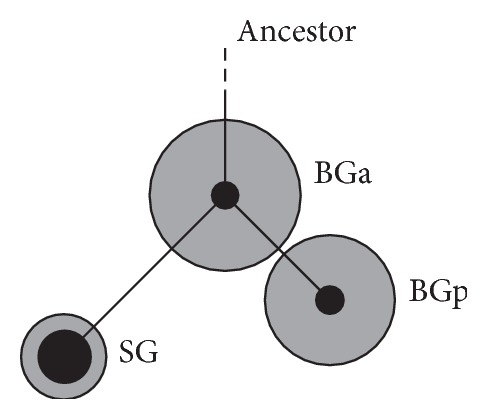
Schematic representation of the network of Baikal oilfish obtained by Teterina et al. [15]. Dark circle diameters are proportional to the frequencies of the main haplotypes of Cytb within the groups and grey circle diameters are proportional to the respective values of nucleotide diversity (*π*).

As previously demonstrated, there are two genetic groups of BG that differ by two nucleotide substitutions in the mitochondrial cytochrome b gene (Cytb) [15]. One group is represented by the line of ancestral haplotypes (BGa) while another is paraphyletically related to BGa (BGp); BGp and SG are derived from the central haplotype of BGa (Figure 1). Nuclear DNA analysis and morphological data revealed no differences between BGa and BGp. It was suggested that these groups correspond to the two peaks of BG spawning. It was also assumed that the differences in mitochondrial DNA are the result of differences in female breeding timing, and nuclear genome panmixia is the result of independent mating of males with females from both genetic groups.

This paper showed signs of selection in synonymous sites of Cytb of Baikal golomyankas directed towards altering the secondary structure of the mRNA and probably altering the character of mtDNA gene expression. We examined the patterns of Cytb molecular variation with the methods of prediction of the secondary structure of nucleic acids and used the data on the fishes' life history.

## 2. Materials and Methods

### 2.1. Sampling and DNA Extraction

Adult specimens were sampled using a beam trawl between 2000 and 2010 in three Lake Baikal basins: southern, central, and northern. Larvae were collected with a large DJOM plankton closing net (2 m diameter, 1 vv mesh) in July, September, and February 2005 to 2011. To determine which of the two spawning groups the BG larvae belonged to, the body lengths of the larvae were measured, which allowed approximate determination of their time of birth. Only individuals with a body length <20 mm were used. Larvae age was approximately assessed as follows: individuals with a body length up to 10 mm were graded as being 1 to 1.5 months old and specimens with a length of 10 to 20 mm were considered to be 1.5 to 3 months old (Elena V. Dzyuba, LIN SB RAS, personal communication). The larvae collected in July and September were attributed to the “summer” spawning peak whereas the larvae collected in February were attributed to the “winter” maximum.

Total DNA was isolated from fish muscle or from whole larvae using phenol-chloroform extraction [18]. An allele-specific real-time polymerase chain reaction (PCR) protocol was developed to identify individuals belonging to a particular genetic group of BG. A similar system was developed to identify BG and SG individuals since species affiliation of larvae and juveniles cannot always be accurately determined based solely on external morphology.

### 2.2. Single Nucleotide Polymorphism (SNP) Analysis

For the rapid and reliable separation of the oilfish individuals by species and groups, SNP markers were designed based on the same DNA sequences as in [15]. The substitutions by which the species and the genetic groups were differentiated are shown in Table 1. To identify the representatives of BG and SG, we used the BG-specific forward primer FBG (5′-ACTACGGATGACTTATCCGTAACC-3′) and the SG-specific forward primer FSG (5′-ACTACGGATGACTTATCCGTAACAC-3′). The reverse primer was common in both cases: RBGSG (5′-TACCCTACGAAAGCGGTTATTATTACAA-3′). To identify the representatives of BGp and BGa, we employed the BGp-specific forward primer FBGp (5′-GCCTGAGTGGTACTTCCTGTTC-3′) and the (BGa + SG) specific forward primer FBGa (5′-GCCTGAGTGGTACTTCTTGTTT-3′). Again, the reverse primer was the same for both: RBGaBGp (5′-CTCCAAGTTTGTTGGGGAT-3′).

Real-time PCR was performed on a Rotor-Gene Q (Qiagen) in a 15 *μ*L reaction mixture containing 1.5 U Encyclo polymerase (Evrogen, Russia), 1.5 *μ*L Encyclo buffer, 1x SYBR Green I dye (BioDye, Russia), 200 *μ*M of each dNTP, 5 pmol of each primer, and 5–50 ng template DNA. The PCR temperature profile involved a 3 min denaturation at 95°C followed by 35 cycles of 10 s at 95°C and 10 s at 60°C. Each reaction was conducted at least in triplicate, and all runs were completed with a melt curve analysis.

### 2.3. Distribution of Mutations

Cytb analyses were performed using largely the same DNA sequences as in [15] supplemented by nine haplotypes of BGp (GenBank Accession Numbers KC571825–KC571833). Mutation calculations were performed according to direction in haplotype genealogy, as evaluated by an unrooted network and a statistical parsimony criterion with NETWORK 4.6 [19] and using the option “statistics.” The distribution of nucleotide diversity (*π*) along Cytb was explored using the Sliding Window Option (SWA) of the program VariScan version 1 [20]. The width of the window was 100 bp, and the window slide was 10 bp.

### 2.4. Prediction of mRNA Secondary Structure

Prediction of mRNA secondary structure (RSSP) was performed using programs UNAFold version 3.8 [16] and RNAfold of the Vienna RNA Package version 1.8.5 [17]. Both synonymous and nonsynonymous mutations were used. A temperature option equal to 3.5°C (which is close to the natural environmental temperature of golomyankas) was chosen to calculate the minimum free energy. To calculate distances between RNA secondary structures, 31 runs of RNAfold and RNAdistance programs [17] were performed with temperatures ranging from 1 to 4°C and steps equal to 0.1°C. The chosen temperature interval roughly covers the range of golomyanka temperature environments. The Fitch-Margoliash method implemented in the Fitch program (PHYLIP package, version 3.6) was used to generate the series of trees based on the distance matrices, and the Consense program (PHYLIP package) was then used to generate a majority rule consensus tree [21]. The values obtained in the internal edges were interpreted as branch support measurements. Sequence analysis, tree drawings, and tree manipulations were performed using MEGA version 6 [22].

## 3. Results

### 3.1. SNP Analysis

The allele-specific primers were tested using DNA samples with known Cytb sequences [15]. In all cases, there was strict conformity between the test results and the nucleotide sequence. We used this approach to determine the ratio of individuals belonging to different genetic groups of BG collected from different regions of Lake Baikal (Table 2). We also analyzed the larvae born in different seasons (Table 3). It was evident that the ratio of individuals belonging to different genetic groups in different basins and in the lake as a whole was close to 1 : 1. Larvae analysis did not reveal any clear patterns between the time of birth and affiliation with a certain genetic group. Representatives of BGa and BGp of the same larval size range were present in each sample.

### 3.2. Mutations Bias

As can be seen in Table 1, the BGa and BGp haplotypes groups differed in two substitutions located at four nucleotides from one another. These were the key substitutions that formed the main haplotypes of the groups. Considering the direction from BGa to BGp (Figure 1), both substitutions were from T to C (T→C). The T→C type polymorphism was also found at position 15500 (data not shown), where the C nucleotide was present at a very high frequency in BGp and absent in BGa. All substitutions were synonymous. Furthermore, we analyzed the amount and distribution of mutations affecting the GC composition of Cytb, which were mutations of weak-AT (W) or strong-GC (S) nucleotides according to their direction from the central to peripheral haplotypes (Test of Centrifugal Substitution Bias, TCSB) within each group (Figure 1, as well as Figure 1 in [15]). To reveal more detailed patterns of mutation distributions within the Comephoridae family, a group of SG haplotypes was also included in the analysis. Nonsynonymous mutations were excluded from the calculations in five sites within BGp and SG and one site within BGa.

**Figure 2:**
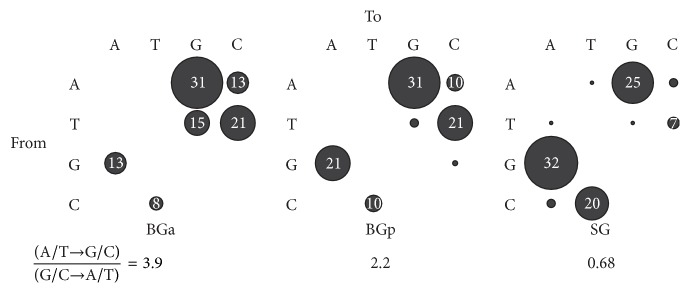
Synonymous mutations in Cytb mapped according to their directions from the central to peripheral haplotypes. Circle sizes and numbers indicate the total number of the mutations as percentages.

**Figure 3:**
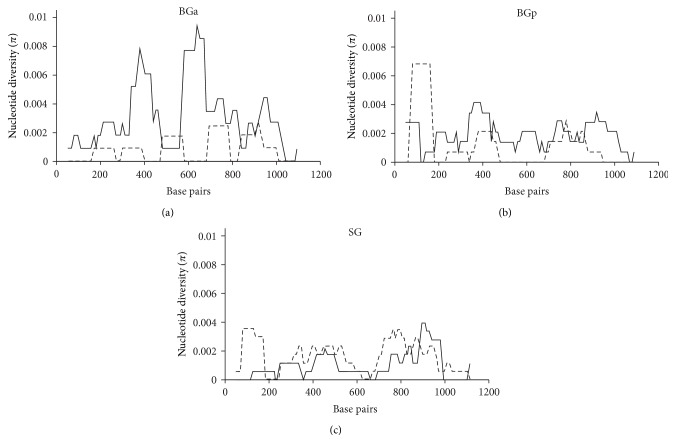
Sliding Window Analysis of nucleotide diversity (*π*) along the Cytb. Bold solid lines indicate W→S mutations and thin dashed lines indicate S→W mutations.

TCSB revealed some differences among the studied groups (Figure 2). The direction of mutations within BGa showed strong deviation toward the W→S type. Within BGp, the predominance of W→S mutations was much weaker. The SG group demonstrated the opposite pattern: the direction to W was prevalent. Fisher's exact test failed to show any significant differences between BGa and BGp but we found statistically significant differences between BGp and SG and very statistically significant differences between BGa and SG (two-tailed *P* values of 0.3073, 0.0147, and 0.0006, resp.). Sliding Window Analysis (SWA) of Cytb showed that, within BGa, W→S mutations dominated over S→W for almost the entire Cytb gene, with two maxima located at ~400 and 600 bp (Figure 3). Thus, the dominance of W→S mutations in this group did not appear to be site- or region-specific. Within SG, the opposite picture was observed; with a few exceptions, S→W mutations dominated. No clear patterns were found within BGp.

### 3.3. RNA Secondary Structure

**Figure 4:**
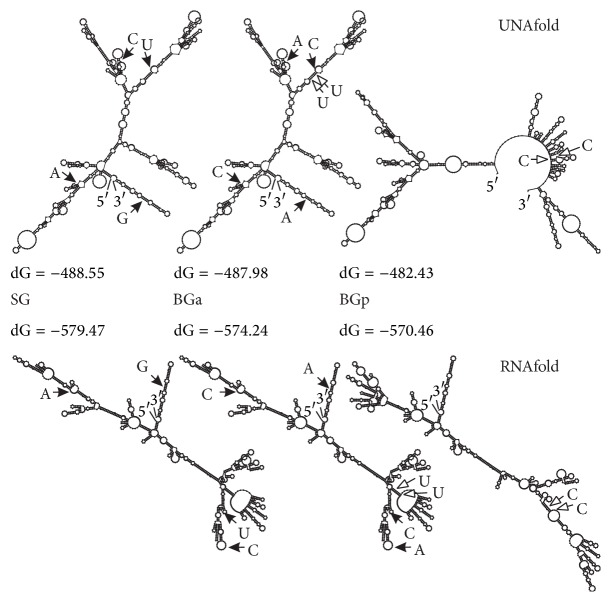
mRNA secondary structure of the main haplotypes of Cytb of BGa, BGp, and SG predicted by UNAFold [16] and RNAfold [17] programs. Black and white arrows indicate substitutions between the main haplotypes of BGa ↔ SG and BGa ↔ BGp, respectively.

As noted above, the main haplotypes of BGa and BGp were separated by the two synonymous substitutions T→C located in close proximity to each other. This probably led to alterations of the local thermodynamic stability of DNA and corresponding mRNA. To test how these substitutions could affect mRNA folding, the secondary structures of mRNA for the main haplotypes of BGa, BGp, and SG were predicted. The mRNA structures predicted by UNAFold and RNAfold were different for the same Cytb sequences. However, both programs showed that the main haplotypes of BGa and SG had very similar structures while the structure for BGp was considerably different (Figure 4).

**Figure 5:**
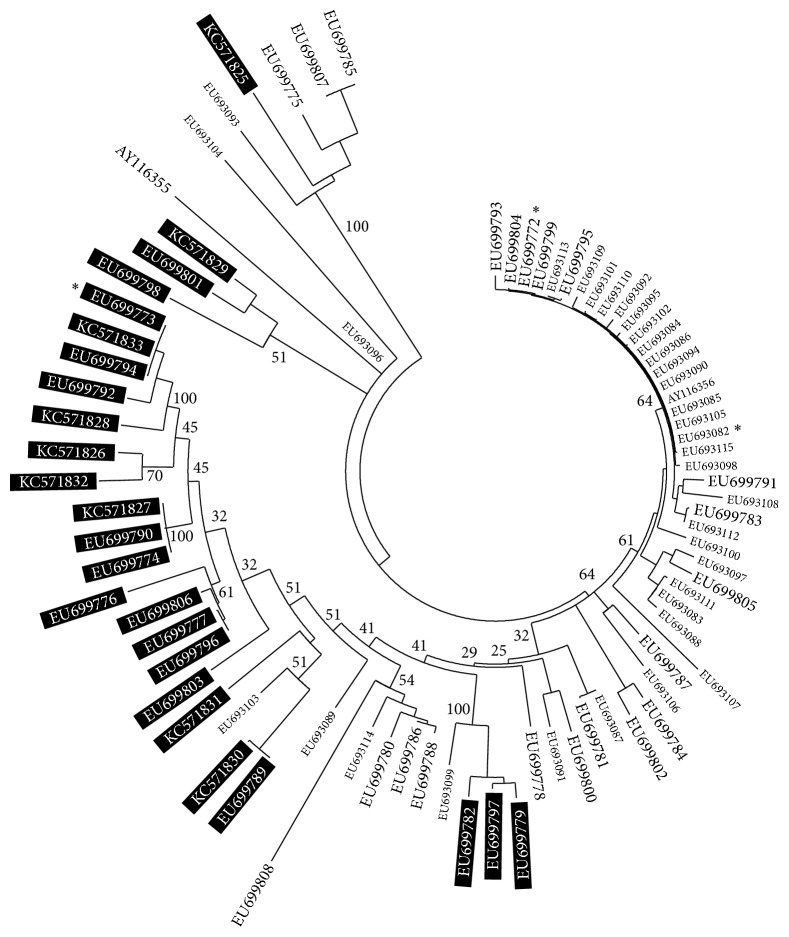
Fitch-Margoliash consensus tree of the Cytb haplotypes of BGa, BGp, and SG based on genetic distances between mRNA secondary structures. Haplotypes are shown as GenBank Accession Numbers. Small letters represent SG; large black letters represent BGa; large white letters in black rectangles represent BGp. Asterisks indicate the main haplotypes. Numbers indicate percentage support values.

According to the tree based on mRNA structures (Figure 5), BGa and SG haplotypes were largely mixed while the representatives of BGp with few exceptions were arranged in a more compact manner: 15 representatives formed one monophyletic group. Although the reliability of the tree was not evaluated in a traditional way and branch support values were not high, its pattern could serve as an additional indirect indicator of a nonrandom distribution of substitutions during the formation of genetic polymorphism of the groups (i.e., the action of selection).

## 4. Discussion

Mutational biases and differences in the nucleotide composition of homologous mtDNA sequences in animals and plants are widely known. Several analyses have been performed and various hypotheses have been proposed to explain these phenomena, but the causes remain unclear [23–25]. The MtDNA of Baikal oilfish can serve as a good example of mutational biases caused by the action of translational selection.

As noted above, very little is known about the posttranscriptional regulation of gene expression in synonymous sites of mtDNA. Many tests and methods used to study these processes in the nuclear genome have been ineffective for mtDNA. Jia and Higgs suggested tests for context-dependent mutation and translational selection in mtDNA [26]. However, these tests could hardly be used for phylogenetically closely related species and groups (e.g., Baikal oilfish), especially when the analysis is restricted to a small DNA fragment (Cytb). The approach used here (TCSB + SWA + RSSP + phylogenetic) was definitely not universal and did not show absolute proof of translational selection in the mtDNA of golomyankas. Nevertheless, there were serious grounds for such assumptions for the following reasons: the distinguishing feature of the studied objects was a close phylogenetic proximity of sympatric species and groups that had similar nucleotide sequence compositions of mtDNA [15, 27] on the one hand and had different vectors of Cytb mutational processes in the different groups on the other hand (Figure 2). One of our results suggested that the distribution of larvae was not consistent with the previously proposed hypothesis of the origin of BGa and BGp representatives from different spawning stock [15] and that the representatives of both BG groups were present in equal amounts in any place within the lake at any time and any age. Taking into account the sympatric character of the formation of intraspecific BG and SG genetic structure and similar demographic characteristics of BGa and BGp [15], the TCSB and SWA differences obtained could not be fully explained by the forces of nonadaptive nature, that is, forces that did not affect the expression of Cytb.

There are several ways of regulating gene expression in synonymous sites associated with mRNA folding, mRNA stability, and mRNA splicing and with preferred synonymous codons [2, 5, 7, 28]. The latter two are apparently not the case in the mtDNA of most animals [26]. The assumption of translational selection was indirectly confirmed by the results of the four tests used here: TCSB, SWA, RSSP, and phylogenetic analysis.

Identified regularities could be associated with the extreme sensitivity of golomyankas to temperature variations [10]. It is known, for example, that the initial stages of thermal alterations in the intensity of oxidative phosphorylation in cardiac muscle cells of Atlantic wolffish occur in enzymatic complex III, which includes the cytochrome b protein [29]. It can be assumed that the synonymous diversity of Cytb within the species and groups of golomyankas could be formed to some extent as a result of the selection pressure of different intensities and directions. We assumed that the genetic diversity within BGa was apparently formed under the action of selection that was directional with respect to the type of mutations against W nucleotides and diversifying with respect to mutation localization (Figure 3). It is possible that selection was associated with the expression of electron transport chain genes by altering the thermodynamic characteristics of the mRNA and consequent refolding. The compactness of BGp on the tree (Figure 5) could indicate an increasing role of a stabilizing selection, which supports a specific mRNA structure. It can be assumed from the two substitutions that separate BGa and BGp made it possible for the representatives of BGp to obtain a partial selective advantage, acting, for example, only under certain environmental conditions so that the total domination of BGp haplotypes within BG did not occur. In that case, the formation of BGp could have happened very quickly in geological time scales and much earlier than the emergence of SG. This could explain some of the discrepancies between the levels of nucleotide diversity within the groups and the number of substitutions between them. For SG and BGp, which have passed through the bottleneck stage and currently have a large population size [9, 15], one would expect a positive correlation to some extent between the distance from the ancestral line (BGa ↔ BGp, BGa ↔ SG) and the magnitude of nucleotide diversity. However, we actually observed an inverse relationship (Figure 1).

One more important result of this work was that golomyankas could serve as a valuable data source for further study of the regulation of mtDNA gene expression and the role of synonymous substitutions in these processes.

## Figures and Tables

**Table 1:** Substitutions, segregating SG, BGa, and BGp, and their location in the Cytb.

Sympatric groups	Substitution positions^*^					
	15627	15966	16004	16206	16211	16502
BGp	**C**	A	C	**C**	**C**	A
BGa	**C**	A	C	**T**	**T**	A
SG	**A**	C	T	**T**	**T**	G

^*^The substitution positions are given relative to the mitochondrial sequence of *Salmo salar* [14] (GenBank Accession Number U12143).

Substitutions used for SNP analysis in bold.

**Table 2:** Number of BGa and BGp adults collected from the south, middle, and north basins of the lake.

Sympatric groups	Basins of the lake			Total number
	South	Middle	North	
BGa	114	19	32	165
BGp	108	18	32	158

**Table 3:** Number of BGa and BGp larvae sampled in the summer (July, September) and winter (February) at height of spawning.

	July	September			February		
Sympatric groups	Length, mm			Total number	Length, mm		Total number
	<10	<10	11–15		11–15	16–20	
BGa	6	8	1	15	3	7	10
BGp	5	7	5	17	6	2	8
